# A randomized clinical trial of the impact of melatonin on influenza vaccine: Outcomes from the melatonin and vaccine response immunity and chronobiology study (MAVRICS)

**DOI:** 10.1080/21645515.2024.2419742

**Published:** 2024-11-13

**Authors:** Rachel U. Lee, Nora L. Watson, Gena L. Glickman, Lindsey White, Sandra D. Isidean, Chad K. Porter, Monique Hollis-Perry, Samuel R. Walther, Santina Maiolatesi, Martha Sedegah, Harini Ganeshan, Jun Huang, David A. Boulifard, Daniel Ewing, Appavu K. Sundaram, Elizabeth M. Harrison, Katherine DeTizio, Maria Belmonte, Arnel Belmonte, Sandra Inoue, Alexandra Easterling, Elizabeth S. Cooper, Janine Danko

**Affiliations:** a Department of Medicine, Walter Reed National Military Medical Center, Bethesda, MD, USA; b Department of Psychiatry and Neuroscience, Uniformed Services University of the Health Sciences, Bethesda, MD, USA; c Henry M. Jackson Foundation for the Advancement of Military Medicine, Inc., Bethesda, MD, USA; d Department of Translational and Clinical Research, Naval Medical Research Command, Silver Spring, MD, USA; e General Dynamics Information Technology, Falls Church, Virginia, MD, USA

**Keywords:** Vaccine immunogenicity, influenza vaccine, melatonin, vaccine adjuvant, humoral immunity, cellular immunity

## Abstract

Vaccine immunogenicity is affected by a variety of factors. Melatonin has been reported to affect immune responses to vaccines and infection. This was a randomized open-label trial – in which adults scheduled to receive the influenza vaccine were randomized to 5 mg melatonin or control to evaluate the effect of post-vaccination melatonin on humoral (hemagglutination-inhibition assays, HAI) and cellular (FluoroSpot) vaccine-specific cytokine responses 14–21 days post-vaccination. A total of 108 participants (melatonin treatment group: 53; control group: 55) completed the study. The groups were similar in baseline characteristics, including sleep as measured by the Pittsburgh Sleep Quality Index. Seroconversion rates or geometric mean fold rises (GMFR) in HAI titers did not vary by treatment group. There were also no statistically significant differences between pre- and post-vaccination levels of interferon gamma (IFN-γ) or granzyme B (GzB) by treatment; however, there was a significantly higher fold rise in the double secretor (IFN-γ + GzB) peripheral blood mononuclear cells for influenza vaccine in subjects taking daily melatonin (GMFR 1.7; 95% CI 1.3, 2.3) compared to those who did not (GMFR 0.9; 95% CI 0.7, 1.1) (*p* < .001). Daily melatonin for 14 days post-influenza vaccination significantly increased the cellular co-expression of IFN-γ + GzB; however, there were no other differences in the cellular or humoral responses. Future studies of the potential utility of melatonin for enhancing vaccine response with larger sample sizes may help elucidate candidate mechanisms for these limited effects, including any interactions with the circadian system.

## Introduction

Vaccinations are a critical component of public health programs that aim to prevent and control the spread of infectious diseases. Yet, despite advances in antigenic targets, vaccine technology, and novel adjuvants, vaccine-induced immunity can be variable.^1,2^ For influenza vaccination, in particular, early predictions about strain characteristics lead to a broad range in effectiveness from year to year; over the past 5 years, the effectiveness of the flu vaccine has ranged from ~10–60%.^3^ In addition, individual differences in intrinsic (e.g., age, sex, genetics, comorbidities) and extrinsic factors (e.g., sleep, exercise, nutrition, site of injection, timing of administration) can further influence vaccination efficacy.^1,2,4^ While some of these mediating factors can be optimized, for others, that may not be possible or practical. Given the high cost of influenza, each year in terms of lost wages, healthcare expenses, resultant post-viral conditions, and other associated burdens and downstream effects, it is critical that to develop ways to enhance the breadth, magnitude, and duration of the immune response induced by the vaccine.^1,2,5^

One putative treatment for enhancing immune response to the influenza vaccine is administration of exogenous melatonin. This hormone has various antioxidant, anti-inflammatory, and anti-excitatory properties, as well as immunoregulatory functions, making the use of melatonin as a vaccine adjuvant an intriguing possibility.^6–8^ Specifically, supplemental melatonin has been shown to enhance vaccine efficacy and has been proposed an adjuvant in animal studies.^9–11^ In in-vitro assays with human peripheral blood mononuclear cells (PBMCs), melatonin promotes humoral immunity.^11^ Melatonin is also known to modulate cell-mediated immunity (CMI); specifically, it stimulates interleukin-2 (IL-2) production, resulting in an increase in CD4+ T cells and IgG-expressing B cells.^12,13^ In older adults, melatonin can mitigate the immunosuppression commonly observed with aging, likely via increased CD4+ lymphocytes.^12^ Interferon gamma (IFN-γ) and granzyme B (GzB) are two important anti-influenza effectors of T cells, and both correlate to influenza illness and vaccination as markers of CMI, though the influence of melatonin on these cells remains unknown.^14,15^ In addition to the more direct effects of melatonin on vaccine efficacy, due to its antioxidant properties and pleiotropic effects on the immune system, treatment with the hormone may also potentially serve to mitigate adverse effects of the vaccine, which may require further investigation.^16,17^

Timing of administration of melatonin is critical as the hormone is both regulated and feeds back on the circadian system. Under normal conditions, there is a nighttime rise in circulating melatonin that is common to both nocturnal and diurnal species, and thus it is sometimes called the “hormone of darkness.” Consequently, in humans, the rise in melatonin is highly synchronized with the typical hours of sleep. Due to its role in sleep and circadian physiology, melatonin may further improve the efficacy of vaccines indirectly via known direct effects on sleep.^18^ Exogenous melatonin has been used in a variety of populations to decrease sleep onset latency and improve sleep efficiency, ultimately increasing total sleep duration.^19^ A recent meta-analysis found that insufficient sleep around the time of vaccination decreases the humoral response against viral vaccinations, though studies to date have generally been small, with variable methods, and different vaccines.^4^ As aspects of the immune system are also under circadian control, modifying sleep, timing of vaccinations, or melatonin may function as “natural adjuvants” and safely improve vaccine immunogenicity.^20–23^

The primary objectives of this study were to evaluate the effects of melatonin on cellular and humoral responses to influenza vaccination. A secondary objective was to explore the role of sleep in observed effects of primary outcomes via both objective and subjective measures; however, these will be published separately. If proven effective as a vaccine or immune adjuvant, there are many advantages to supplemental melatonin from a practical standpoint. Melatonin has a very high safety profile, can be easily self-administered, and is relatively inexpensive.^24^ In the US, it is sold as an over the counter (OTC) supplement, which makes it readily accessible, but also results in significant variability in quality and dosing.^25^ Currently, human data are inadequate to support the widespread use of melatonin to improve vaccine immunogenicity, or for other immune benefits.^6,7,12^ Given the current literature and gaps in knowledge regarding melatonin and vaccine immunogenicity, we sought to study the effects of daily administration of exogenous melatonin on humoral and cellular responses following influenza vaccination.

## Methods

### Study design

This was a randomized, open-label pilot trial designed to evaluate the effect of melatonin and sleep on influenza vaccine-induced immunogenicity. Participants were enrolled from October 2022 to January 2023 at the Walter Reed National Military Medical Center (WRNMMC) and the Naval Medical Research Command (NMRC) Clinical Trials Center in Bethesda, MD. The protocol (NMRC.2021.0006) was approved by the NMRC Institutional Review Board in compliance with all Federal regulations governing the protection of human subjects and registered on clinicaltrials.gov (NCT04953754).

Participants were screened for eligibility and underwent a general health screening after providing written informed consent. Healthy adults aged 18–64 years who were eligible and planning to receive influenza vaccination at WRNMMC were enrolled. Exclusion criteria included receipt of influenza vaccine within the previous 6 months prior to enrollment, an allergy or contraindication to influenza vaccine(s), medical history of an immune-compromising medical condition (i.e., HIV, active cancer, etc.), a diagnosed sleep disorder requiring medication (i.e., insomnia, narcolepsy, etc.), pregnancy, or recent history (within 3 months) of taking immunosuppressant or immune-modifying treatment(s) (i.e., systemic corticosteroids, chemotherapy, IVIG, blood transfusion, etc.), and/or use of supplements, or medications prescribed, for sleep in the past month. Older participants were not included as they would have received the high-dose or adjuvanted influenza vaccines. All inclusion and exclusion criteria were evaluated by one or more of the study’s physicians to determine eligibility.

At visit 1, prior to influenza vaccination and following an informed consent process, participants were randomized one-to-one (with random block sizes of 4 and 6) to receive either melatonin (treatment) or no melatonin (control). Participants also provided a blood sample and completed a study questionnaire with the following items: demographic information, the Pittsburgh Sleep Quality Index (PSQI), and the Munich Chronotype Questionnaire. Due to the large volume and complexity of this study dataset, a comprehensive report of the latter two validated assessments, along with objective measures of sleep (actigraphy) and immunogenicity, will be included in a second publication; however, to establish similarity between groups, total sleep time reported at enrollment is included in the analysis of baseline differences reported here. Participants were then advised to go to the WRNMMC immunization clinic to receive inactivated influenza vaccination (IIV4) (FluLaval® Quadrivalent, GSK, Quebec, Canada) within 24–48 h. Visit 2 occurred 14–21 days after the initial visit, during which participants provided another blood sample and completed a second study questionnaire.

### Antigens

The influenza strains included in the 2022/23 IIV4 vaccine that the enrolled subjects received included: A/Victoria/2570/2019 (H1N1), A/Darwin/9/2021 (H3N2), B/Austria/1359417/2021 (B/Victoria lineage), and B/Phuket/3073/2013 (B/Yamagata lineage).^26^

### Treatment

Participants randomized to the treatment group were given REMfresh® 5 mg caplets (Nestle Health Science), an OTC melatonin supplement as recommended by a board-certified sleep physician consultant at WRNMMC. Participants assigned to the treatment group were instructed to take a single caplet 1 h before their planned bedtime for 14 days, starting on the night of influenza vaccination. High-Performance Liquid Chromatography (HPLC) was performed at an independent lab (NSF Laboratories, Ann Arbor, MI) to quantify the amount of melatonin per caplet. Five caplets from the lot used in the study were tested and contained between 5.5 and 6.1 mg of melatonin.

### Immunological assessments

#### Serology

Influenza-specific serum antibodies were quantified by hemagglutination-inhibition (HAI) assays using standard procedures in blinded samples. Briefly, sera were treated at a 1:3 ratio (vol/vol) with receptor-destroying enzyme (RDE) at 37°C for 18–20 h to eliminate nonspecific inhibitors of agglutination. RDE was subsequently inactivated by incubation at 56°C for 45 min, followed by the addition of six volumes of phosphate-buffered saline (PBS) resulting in an initial testing dilution of 1:10. All RDE-treated sera were tested for nonspecific agglutinins, and positive sera were heme-adsorbed using turkey erythrocytes prior to performing the HAI assay; each sample was tested in duplicate.^27^

Influenza virus standardized to eight hemagglutinin units (HAU) per 50 μL (4HAU per 25 μL) in PBS were added to two-fold serial dilutions of test sera. Following incubation at room temperature for 30 min, 0.5% turkey red blood cells were added. Plates were observed for agglutination after 30 min. The HAI titer was defined as the reciprocal of the highest dilution of serum that completely inhibited hemagglutination. The geometric mean titer (GMT) was calculated for each sample duplicate and reported as the final titer. For computational purposes, titers of <1:10 were assigned a value of 1:5. Pre- and post-vaccination serum samples were processed and tested together.^27^

#### Cell-mediated immunity (IFN-γ and GzB)

Blinded heparinized whole blood samples were collected, sent to the lab, and PBMCs were separated at baseline and 14–21 days post-vaccination. PBMCs were stored in liquid nitrogen until use. Samples from both visits were run concurrently in duplicates. PBMCs were stimulated in vitro with different types of stimulants at various final concentrations. The stimulants included whole vaccine product (FluLaval® Quadrivalent, GSK, Quebec, Canada) at 0.25 µg/mL and 1 µg/mL, Flu Class I peptide pool (C.T.L., Shaker Heights, Ohio), used at 1 µg/mL, and PepMix Influenza A (JPT Peptide Technologies, Berlin, Germany), a mixture of peptides derived from antigenic components from Influenza A, and used at 1 µg/mL. Negative controls consisted of cells without antigenic stimulant, and PHA, a mitogen, was used as a positive control for cell viability. PBMCs were tested at 400 K (for peptide pool stimulation), and 200 K/well (for FluLaval® stimulation). Antigen-specific cells secreting cytokines were recognized as spots, enumerated as spot forming cells/well (sfc/w), converted and reported as spot forming cells/million (sfc/m) PBMC.

Responses to a specific stimulant were also reported as positive or negative using pre-defined criteria as follows: (1) the stimulation index, defined as the ratio between average of antigen-specific stimulated sfc/well and unstimulated sfc/well was greater than or equal to (≥) 2, and (2) the difference of the average antigen-specific stimulated sfc/well – from the unstimulated sfc/well was greater than (>) 10 sfc/well.

FluoroSpot kits (Mabtech AB, Nacka Strand, Sweden) were used according to manufacturer’s directions. This is an enhanced form of ELISpot assay based on fluorescent labeling, enabling detection of single cells secreting multiple cytokines. The assay is highly sensitive which allows for the monitoring of low frequencies of antigen-specific cytokine secreting cells and/or effector molecule secreting cells after vaccination or infection. Upon antigen-specific stimulation, the assay can detect single cells in the same well that secrete single cytokine or effector molecules (e.g., IFN-γ, GzB), or single cells secreting multiple cytokines and/or effector molecules in different combinations that include IFN-γ+GzB (double-secretor) and others.^28–30^

#### Statistical analysis

Seroconversion rates were defined *a priori* as the percent of subjects with a pre-vaccination titer <1:10 who had a post-vaccination titer ≥1:40, or with a pre-vaccination titer ≥1:10 who had a four-fold rise or higher titer post-vaccination.

Based on prior studies, we anticipated seroconversion rates between 20% and 70% in the control arm.^31^ Based on this estimate, between-group comparisons with 100 subjects per group would have ≥80% power (two-sided alpha = 0.05) to detect a clinically relevant absolute difference in seroconversion rates of ≥20%.

Descriptive statistics were reported for baseline demographic characteristics and total sleep time at enrollment. Geometric mean titers (GMT) and 95% confidence intervals (CIs) were estimated at baseline and post-vaccination by study group. Geometric mean fold rises (GMFR) and 95% CIs were calculated to compare post-vaccination to baseline responses in each group. CIs for GMT and GMFR were calculated based on the t-distribution of the log-transformed responses and back transformed to their original scale. Rates of seroconversion for each group are reported with 95% exact CIs.

Post-vaccination GMT and GMFR were compared between groups using two-sample t-tests of the log-transformed responses. Seroconversion rates were compared between groups using Chi-squared or Fisher’s exact tests. All statistical tests were evaluated at alpha = 0.05 with all effect sizes and their 95% CIs reported. Analyses were performed using R version 4.0.5.^32^

The randomization sequence was generated by the statistician using R (Vienna, Austria) with a 1:1 treatment to control allocation with random block sizes of 4 and 6. Treatment was allocated to a subject ID assigned in order of consenting and meeting inclusion and no exclusion criteria.

## Results

### Participant characteristics

**Figure 1.**
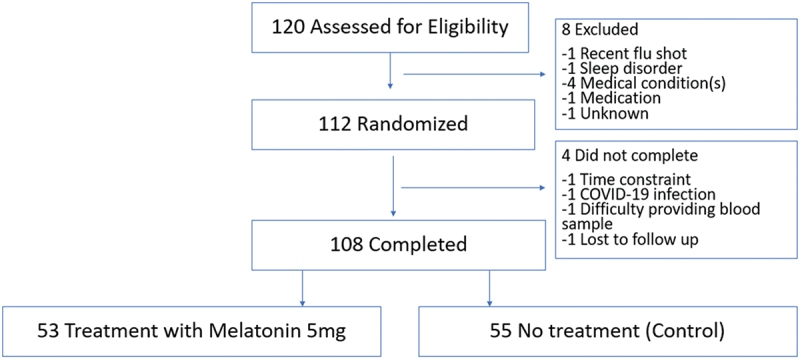
Flow diagram of eligibility and follow-up (separately attached).

Among the 120 participants screened, 112 were randomized, enrolled, and received randomly assigned product. Between the first and second visits, three subjects withdrew, and one was lost to follow-up (Figure 1). Reasons for withdrawal included time constraints, COVID-19 infection, and difficulty providing a blood sample. Of the 108 participants who completed the study, 50.9% were female, all were between 18 and 64 years of age (mean 38 ± 12 yrs) and 50.9% White; 53 were randomized to the treatment (melatonin) and 55 to control (no melatonin) groups (Figure 1). All 108 participants were included in all analyses. Baseline demographic characteristics were similar between the two groups (Table 1). Total sleep times were also similar: 6 h 41 min ±58 min and 6 h 36 min ±71 min for the treatment and control groups, respectively). The average time of reported melatonin ingestion was 57 min prior to sleep time, consistent with instructions.

**Table 1.** Characteristics of the study participants at baseline.

Characteristic	Treatment (melatonin)	Control (no melatonin)
*N* = 108	53	55
Sex; n (%)		
Female	30 (56.6%)	25 (45.5%)
Male	23 (43.4%)	30 (54.5%)
Age in years; mean (std dev)	38.0 (12.1)	39.0 (12.3)
Ethnicity*; n (%)		
Hispanic	9 (17.0%)	3 (5.5%)
Non-Hispanic	35 (66.0%)	43 (78.2%)
Unknown/Unreported	9 (17.0%)	9 (16.4%)
Race*; n (%)		
Asian	3 (5.7%)	5 (9.1%)
Black/African American	16 (30.2%)	18 (32.7%)
American Indian/Alaskan	1 (1.9%)	1 (1.8%)
Other	6 (11.3%)	2 (3.6%)
White	26 (49.1%)	29 (52.7%)
Not Reported	1 (1.9%)	0 (0.0%)
Active-Duty Military; n (%)		
Yes	22 (41.5%)	30 (54.5%)
No	31 (58.5%)	25 (45.5%)

*Self-reported.

### HAI seroconversion rates

The HAI seroconversion rates ranged from 7.3% to 64.2%, depending on the study group and the strain tested, with no significant differences between the two groups. The highest response rates were observed against the A/Darwin/9/2021 (H3N2) strain followed by B/Austria/1359417/2021 (Table 2). Notably, the higher response rates were to the two new strains included in the 2022/23 vaccine formulation. There were no differences between the pre- and post-vaccination titers in the treatment vs. control groups (Table S1, Supplementary Material).

**Table 2.** HAI seroconversion rates (95% CIs) by the treatment group.

Influenza strain	Melatonin (*n* = 53)	Control (*n* = 55)	*p*-value
A/Victoria	9.4% (3.1, 20.7%)	14.5% (6.5, 26.7%)	.56
A/Darwin	64.2% (49.8, 76.9%)	56.4% (42.3, 69.7%)	.44
B/Austria	30.2% (18.3, 44.3%)	30.9% (19.1, 44.8%)	>.99
B/Phuket	11.3% (4.3, 23.0%)	7.3% (2.0, 17.6%)	.52

Footnotes: CIs, confidence intervals; strains tested are as follows: A/Victoria/2570/2019 (H1N1), A/Darwin/9/2021 (H3N2), B/Austria/1359417/2021 (B/Victoria lineage), and B/Phuket/3073/2013 (B/Yamagata lineage).

### Cellular (FluoroSpot) responses

There were no significant differences in the geometric mean FluoroSpot responses between treatment and control groups post-vaccination (Table S2). However, the geometric mean fold rise (GMFR) in the double secreting PBMCs (IFN-γ+GzB) was significantly higher in the melatonin treated vs. control group at the 1 µg/mL final concentration of FluLaval® (Table 3) (GMFR 1.7 (95% CI 1.3, 2.3) vs 0.9 (0.7, 1.1) *p* < .001). While multiple dilutions of FluLaval® were tested, lower concentrations were suboptimal and did not reach statistical significance.

**Table 3.** Geometric mean fold rises (95% CI) in fluorospot responses by the treatment group.

Test	Melatonin Treatment (*n* = 53)	Control (*n* = 55)	*p-*value
FluLaval 1ug/mL			
IFN-γ	1.4 (1.3, 1.6)	1.4 (1.2, 1.5)	.42 .59
GzB	2.1 (1.2, 3.5)	1.4 (0.9, 2.1)	
IFN-γ+GzB	1.7 (1.3, 2.3)	0.9 (0.7, 1.1)	<.001

## Discussion

This is the first clinical trial to prospectively evaluate the effect of supplemental melatonin on both the humoral and cellular immune responses to vaccination. We saw no significant effect of melatonin on the humoral immune responses to influenza vaccination by HAI, the accepted correlation of protection for influenza. CMI, specifically T cell activity, may serve as a marker of antiviral response against infection and vaccination.^14^ Although there was no statistically significant difference in the levels of individual cytokine response to influenza vaccine, we did observe an increase in the PBMC’s secreting *both* IFN-γ and GzB in melatonin-treated compared to untreated participants. This finding is supported by in vitro studies showing that melatonin enhances the production of IFN-γ and GzB which may lead to a more coordinated efficient and effective anti-viral response, and less of a single cytokine-driven immune or inflammatory response.^33–35^ IFN-γ has multiple effects on innate and adaptive immunity and inflammation.^35^

Hyperinflammation can result from overproduction of IFN-γ with downstream pro-inflammatory cytokines, such as tumor necrosis factor (TNF)a, interleukin (IL)-1, IL-18, and others; studies have demonstrated serious hyperinflammatory and immune-mediated diseases, which include autoinflammatory diseases, cytokine storm, and other.^35^ Given the potential for deleterious effects of excess inflammation and clinically beneficial correlation of higher IFN-γ and GzB levels with clinical influenza infection and post-vaccination, these data warrant further research.^15,35–38^

We evaluated a single OTC dose of melatonin. Although our product was assayed and found to contain approximately 5 mg of melatonin, OTC supplements such as melatonin are not subject to the same regulations as pharmaceuticals in the UK and Canada. As such, there may be variability in active melatonin and other contaminants in some products.^25^ Higher doses of melatonin (up to 100 mg) have been found to be safe and could conceivably be evaluated in future studies.^39^ The effects of melatonin may also vary by sex and weight; these parameters were not assessed in our study.

Our results should be interpreted with a full appreciation of the inherent limitations. First, this was not a double-blind, placebo-controlled study, and participants were aware of their group assignment; however, this awareness is not expected to affect the primary immunology outcomes. We did not reach our target enrollment due to a decreasing number of interested participants that had not yet received their annual influenza vaccine and analyses were underpowered to demonstrate statistically significant differences for modest treatment effects. Nonetheless, HAI titers between groups were comparable and it is unclear if significant differences would have been clinically meaningful. It should be noted that because the COVID-19 pandemic was ongoing during this study and, some subjects opted to receive a COVID-19 booster concurrently with their influenza vaccine. This may have modulated immune response to the influenza vaccine.^2^ Data on vaccine co-administration were not collected and we are unable to assess this potential effect. Participants initiated melatonin on the day of influenza vaccination; however, the optimal timing and dose of supplementation relative to vaccination is unknown.^2,40^ Our population was limited to adult participants and excluded older (≥65 years of age) and younger (<18 years of age) participants in whom melatonin may have different effects; in particular, it is unknown if melatonin would have a different immunologic impact on higher dose or adjuvanted vaccine in older populations with lower levels of endogenous melatonin.^2^ We did not assess vaccine effictiveness limiting the clinical interpretation of our findings. There are numerous other potential unmeasured confounders (e.g., stress, exercise, depressed mood, weight, smoking, alcohol, etc.) that may have affected the vaccine immune responses in our study. However, the strength of the design and randomized treatment allocation was shown to achieve a balance between treatment groups in age and other key demographics and total sleep time, mitigating risk of substantial unmeasured confounding that may be considered to potentially bias comparisons of immune responses by treatment group. In addition to sleep and the circadian system, IFN-γ and GzB, along with most cytokines, follow circadian rhythms and optimizing timing of vaccination and sample collection to measure these parameters may require further study.^2,31,40–43^ Finally, the ability to modulate sleep naturally or with supplementation warrants additional study,^2,31,44,45^ and this will be examined separately within our dataset.

## Conclusion

In summary, daily melatonin (5 mg) supplementation did not significantly increase humoral or cellular responses to influenza vaccination in this study. However, there was a significantly higher GMFR for IFN-γ + GzB response in the melatonin treated group, warranting further investigation. Annual influenza epidemics continue to result in significant morbidity and mortality worldwide.^40,42^ Given the importance of vaccination for primary prevention of influenza and many other infections, further studies of modifiable and safe factors to enhance vaccine-induced immunity are critical.

## Supplementary Material

Supplemental Material

## Data Availability

Data are subject to an ethical restriction for personal data. Any requests for underlying data should be directed to: Chad K. Porter, Naval Medical Research Center, Silver Spring, MD; chad.k.porter2.civ@mail.mil
