# Supplementary material for: A randomized clinical trial of the impact of melatonin on influenza vaccine: Outcomes from the melatonin and vaccine response immunity and chronobiology study (MAVRICS)
Source: Hum Vaccin Immunother. 2024 Nov 13;20(1):2419742. doi: 10.1080/21645515.2024.2419742 (PMC11572083; doi:10.1080/21645515.2024.2419742)
Supplement: Supplemental Material [file KHVI_A_2419742_SM6796.pdf]

SUPPLEMENTARY MATERIALS

Table S1. Geometric mean HAI antibody titers by treatment group and time.

	**Melatonin (n=53)**		**Control (n=55)**		**P value***
Test	Prevaccination	Postvaccination	Prevaccination	Postvaccination	
A_Vic	130.6 (95% CI 106.3, 160.6)	193.4 (95% CI 161.2, 232.1)	147.4 (95% CI 117.5, 184.9)	205.9 (95% CI 169.9, 249.5)	0.64
A_Dar	16.3 (95% CI 11.9, 22.5)	80.0 (95% CI 58.9, 108.6)	16.0 (95% CI 11.8, 21.8)	77.0 (95% CI 56.1, 105.9)	0.86
B_Aus	11.9 (95% CI 9.3, 15.1)	32.7 (95% CI 23.2, 45.9)	14.8 (95% CI 10.6, 20.6)	38.8 (95% CI 27.3, 55.1)	0.48
B_Phu	10.3 (95% CI 8.3, 12.8)	15.6 (95% CI 12.0, 20.3)	9.3 (95% CI 7.5, 11.5)	13.5 (95% CI 10.6, 17.3)	0.43

*Postvaccination

Table S2. Geometric mean fluorospot responses by treatment group and time.

	**Melatonin (n=53)**		**Control (n=55)**		**P value***
Test	Prevaccination	Postvaccination	Prevaccination	Postvaccination	
Flu Class I					
IFN-γ	3.0 (95% CI 1.5, 5.8)	3.4 (95% CI 1.8, 6.7)	2.4 (95% CI 1.2, 4.8)	2.8 (95% CI 1.5, 5.2)	0.65
GzB	2.3 (95% CI 1.3, 3.9)	1.9 (95% CI 1.1, 3.5)	1.9 (95% CI 1.1, 3.5)	1.3 (95% CI 0.7, 2.2)	0.31
IFN-γ +GzB	0.3 (95% CI 0.2, 0.4)	0.3 (95% CI 0.2, 0.5)	0.3 (95% CI 0.2, 0.4)	0.2 (95% CI 0.2, 0.4)	0.34
FluLaval 1ug/mL					
IFN-γ	350.8 (95% CI 302.8, 406.3)	465.4 (95% CI 409.0, 529.4)	382.9 (95% CI 331.4, 442.4)	479.4 (95% CI 419.3, 548.1)	0.75
GzB	26.8 (95% CI 14.4, 50.1)	40.1 (95% CI 23.4, 68.6)	45.0 (95% CI 27.8, 72.9)	53.9 (95% CI 35.2, 82.4)	0.39
IFN-γ +GzB	14.1 (95% CI 8.8, 22.6)	22.2 (95% CI 15.4, 31.8)	23.2 (95% CI 16.8, 32.1)	17.4 (95% CI 11.3, 26.9)	0.40

*Postvaccination
